# The Mediating Role of Emotion Dysregulation and Hopelessness in the Association Between Attachment and Meaning in Life

**DOI:** 10.3390/bs16020242

**Published:** 2026-02-09

**Authors:** Xavier Sebastián Sanz Sendra, Sandra Pérez Rodríguez

**Affiliations:** 1 Departamento de Psicología Básica, Neuropsicología y Social, Universidad Católica de Valencia “San Vicente Mártir”, 460100 Burjassot, Spain; 2 Department of Personality, Assessment and Psychological Treatments, Universidad de Valencia, 46010 Valencia, Spain; sandra.perez@uv.es

**Keywords:** meaning in life, attachment, hopelessness, emotion dysregulation, Spanish adolescents

## Abstract

Meaning in life (MIL) is recognized as a protective factor for adolescent mental health, being associated with reduced risks of depression, suicidal ideation, and non-suicidal self-injury. This study examined associations between attachment styles and MIL, with hopelessness and emotion dysregulation as potential mediators. Participants were 2067 Spanish adolescents (51.9% male, 48.1% female; M age = 14.62, SD = 1.80) who completed validated measures of attachment, emotion dysregulation, hopelessness, and MIL. Structural equation modeling indicated that secure attachment was associated with higher MIL both directly and indirectly through lower levels of hopelessness and emotion dysregulation. Disorganized attachment was not directly associated with MIL but was indirectly associated with MIL through these vulnerabilities. Hopelessness emerged as the strongest mediator, while emotion dysregulation was associated with satisfaction and meaning only. Findings highlight psychological vulnerabilities as pathways linking attachment to MIL, with implications for clinical and preventive strategies.

## 1. Introduction

Although debates persist regarding its conceptual boundaries (King & Hicks, 2021) and concerns have been raised about the overlap of meaning in life (MIL) scales with constructs such as affect and life satisfaction (Steger et al., 2006), MIL is generally understood as the perception of one’s existence as purposeful, goal-directed, and embedded within a broader framework of significance (George & Park, 2016). From a logotherapeutic perspective, Frankl (1988) emphasized that the conviction that life has meaning constitutes a fundamental human motivation, providing both a framework for interpreting experience and a psychological resource that fosters resilience, goal pursuit, and well-being.

Contemporary models conceptualize MIL as comprising three dimensions: coherence, or the sense that life is comprehensible and structured; purpose, defined by the pursuit of valued, future-oriented goals; and significance, referring to the belief that life is valuable and worthwhile (Martela & Steger, 2016). Empirical findings, though largely correlational, consistently link higher MIL to lower engagement in health-risk behaviors (Brassai et al., 2011), reduced prevalence of non-suicidal self-injury (Gallego-Hernández de Tejada, 2019; Marco et al., 2020), and a buffering effect against interpersonal and cognitive vulnerabilities (Marco et al., 2015; Zeng et al., 2024). Elevated MIL is further associated with greater psychological resilience, reduced psychiatric symptoms, lower rates of suicidal ideation, and enhanced social and economic resources (Boyle et al., 2009; Hill et al., 2016; Routledge & FioRito, 2021; Yu & Chang, 2021).

Considering its established links to mental health, increasing attention has been paid to understanding why some individuals report stronger perceptions of coherence, purpose, and significance than others (Guenther, 2025; King & Hicks, 2021). Attachment theory provides a central framework for understanding these processes. Bowlby (1973) argued that early caregiver–child interactions give rise to internal working models—cognitive–affective schemas that shape perception, emotion regulation, and behavior. Crucially, he did not conceptualize these models as static or neutral reflections of reality but as dynamic structures influenced by emotional evaluations and motivational orientations, situated within relational contexts (Bretherton & Munholland, 2008). Bowlby’s formulation was inspired by Craik’s (1943) proposal that humans actively construct mental models of the world to anticipate events, plan actions, and adapt flexibly to environmental demands, with thinking understood as the manipulation of such representations (Johnson-Laird, 1983). In this vein, Bowlby (1979), in his seminal essay “On Knowing What You Are Not Supposed to Know and Feeling What You Are Not Supposed to Feel”, argued that early interactions with caregivers can invalidate children’s perceptions, censure their emotional responses, or inhibit recognition of aspects of their own personality. Such pressures—made effective by the child’s need for love and protection—thereby shape the development of internal working models. More recently, Dewitte et al. (2019) argued that, although humans may possess an innate propensity to construct meaning, attachment experiences shape this process by organizing cognitive and relational representations from early childhood, thereby laying the foundation for perceiving life as purposeful and valuable.

Building on Bowlby’s conceptualization, Ainsworth et al. (1978) empirically identified three primary attachment patterns—secure, avoidant, and ambivalent—which were later expanded by Main and Solomon (1986) to include disorganized attachment. Subsequent research, including a growing body of longitudinal and cross-sectional studies, indicates that sensitive and responsive caregiving fosters secure attachment representations and adaptive emotional regulation, whereas neglectful or abusive experiences heighten the risk of insecure or disorganized attachment (Cassidy et al., 2018; Mikulincer & Shaver, 2016). Such disruptions have enduring implications for stress regulation, interpersonal adjustment, and, ultimately, the individual’s capacity to construct and sustain a coherent sense of meaning. In line with this perspective, Mikulincer and Shaver (2013) argue that attachment security constitutes a critical basis for perceiving life as coherent, valuable, and imbued with authentic meaning. Grounded in positive relational experiences, such security not only fosters the capacity to view life as meaningful but also facilitates the development of supportive interpersonal bonds and the pursuit of goals that are both consistent and adaptive.

Hopelessness is conceptualized as a cognitive distortion characterized by the perception of a lack of personal control over future events and by expectations of failure or adverse outcomes (Beck et al., 1974). Considered a transdiagnostic construct, it has been identified as a key factor underlying a wide range of mental health disorders (Beck & Steer, 1988). More recently, Sukma and Puspitasari (2023) described hopelessness as a pervasive sense of helplessness that erodes vitality, diminishes hope, and reduces aspirations, often arising from insufficient social support. Within this framework, hopelessness fosters feelings of fatigue and discouragement, which in turn hinder individuals’ capacity to engage in effective problem-solving. Existing literature consistently links insecure attachment with elevated levels of hopelessness (Ringer et al., 2014). Similarly, elevated levels of hopelessness are inversely associated with MIL (Sun et al., 2022).

Emotion regulation refers to the set of processes through which individuals monitor, modify, and express their emotional responses, shaping their onset, intensity, duration, and offset (Gross et al., 1998). Dysregulation may emerge from the absence of regulation attempts, the ineffective implementation of strategies, or the reliance on maladaptive mechanisms (Wolff et al., 2019). Gratz and Roemer (2004) delineated six domains in which regulatory difficulties may arise: limited awareness and clarity of emotions, non-acceptance of emotional experiences, restricted access to perceived effective strategies, difficulties controlling impulses when distressed, and impairments in goal-directed behavior under negative affect. Complementing this framework, Hasking et al. (2017) emphasize the role of heightened emotional reactivity and attentional biases—shaped by temperament or early life experiences—in fostering a preferential focus on negative stimuli and the consolidation of maladaptive self-schemas. Attachment theory posits that the attachment system regulates distress by activating proximity-seeking strategies toward protective figures (Main, 1990). In adulthood, this emotional regulation occurs through direct support seeking, internalized representations, or self-soothing routines, highlighting attachment’s evolutionary role as a core mechanism for managing negative affect in a social species (Mikulincer & Shaver, 2008). Moreover, Lin (2022), in a study with 1312 participants, found that emotion regulation strategies significantly influence students’ MIL. Similarly, Chen et al. (2022) reported that emotion regulation moderates the relationship between MIL and mental health.

For this reason, both hopelessness and emotion dysregulation were included in the model, as outlined above, to capture complementary mechanisms linking attachment to MIL. Specifically, hopelessness represents a future-oriented cognitive vulnerability that constrains purpose and goal pursuit (Beck et al., 1974; Sun et al., 2022), whereas emotion dysregulation reflects difficulties in managing emotional experiences that limit the integration of affect into coherent meaning (Gross et al., 1998; Gratz & Roemer, 2004; Lin, 2022). From an attachment perspective, internal working models are theorized to shape both future expectations and emotion regulation strategies (Bowlby, 1973; Mikulincer & Shaver, 2008), supporting the inclusion of both pathways.

Accordingly, we hypothesize that hopelessness and emotion dysregulation operate as proximal mechanisms linking attachment to MIL. Although prior research has examined associations among these constructs, to the best of our knowledge no study has directly investigated the mediating role of hopelessness and emotion dysregulation in this relationship. The present study aimed to address this gap by pursuing two objectives: (a) to examine the associations among attachment styles (secure and disorganized/traumatic), emotion dysregulation, hopelessness, and MIL in Spanish adolescents; and (b) to investigate whether attachment styles are indirectly associated with MIL—satisfaction and meaning and vital goals and purposes—through their associations with emotion dysregulation and hopelessness.

## 2. Method

### 2.1. Participants

The study sample comprised *N* = 2067 adolescents enrolled in secondary education across three Spanish regions: Madrid, the Valencian Community, and the Basque Country. Participation in the study was voluntary and required written parental informed consent as well as adolescents’ assent. Not all eligible students from the participating schools took part in the study, primarily due to the absence of parental authorization. The exact proportion of the targeted student population could not be determined, as participating schools did not provide the total number of enrolled students. Reasons for non-participation included parental concerns regarding the assessment of non-suicidal self-injury, lack of parental consent, and voluntary withdrawal by some adolescents despite having obtained parental authorization. Recruitment was conducted between February and March 2022 through collaborations with a national network of charter schools. Although institutions in nine autonomous communities were contacted, only five schools agreed to participate—three located in Madrid, one in the Basque Country, and one in the Valencian Community. As a result, the sampling procedure was non-probabilistic and based on convenience, yet it yielded representation from northern, eastern, and central Spain, ensuring geographical variability despite its limitations.

In terms of nationality, the sample was composed of Spanish-born participants (98.6%, *n* = 2027), with a minority of students (1.4%) originating from other countries across Europe, the Americas, Africa, and Asia (e.g., Andorra, Argentina, Cameroon, China, the United States, Italy, Mexico, Ukraine, and Vietnam).

The sex distribution was nearly balanced, with 51.9% boys (*n* = 1072) and 48.1% girls (*n* = 995). The age range extended from 11 to 19 years (*M* = 14.62, *SD* = 1.80). The largest subgroups were concentrated between 12 and 16 years: 14.8% aged 12, 17.2% aged 13, 16.9% aged 14, 15.9% aged 15, and 16.3% aged 16. Smaller proportions were observed at the extremes of the distribution, with only 2 participants aged 11 (0.1%) and 14 participants aged 19 (0.7%). Two respondents did not provide age data.

Regional representation was uneven, with the Community of Madrid accounting for the majority (64.6%, *n* = 1335), followed by the Basque Country (22.6%, *n* = 467) and the Valencian Community (12.8%, *n* = 265).

### 2.2. Instruments

Attachment representations were assessed using the Cartes: Modèles Individuels de Relation—Revised (CaMir-R; Pierrehumbert et al., 1996; Spanish adaptation: Balluerka et al., 2011), a 32-item instrument validated in Spanish adolescents and adults. Following Lacasa and Muela’s (2014) proposal, four dimensions were examined in this study: Security, indexing confidence in caregivers and perceptions of consistent love and support (secure attachment); Parental Interference, reflecting memories of childhood overprotection and fear of abandonment (preoccupied attachment); Self-sufficiency and Resentment toward Parents, capturing emotional distancing, rejection of dependence, and hostility toward caregivers (avoidant attachment); and Childhood Trauma, encompassing recollections of neglect, violence, or threat from attachment figures (disorganized/insecure attachment). Responses are provided on a 5-point Likert scale. The CaMir-R has demonstrated adequate psychometric properties in prior research (Balluerka et al., 2011). In the present sample, internal consistency was adequate for the Security (α = 0.87) and Childhood Trauma (α = 0.78) subscales, but lower for Parental Interference (α = 0.61) and Self-sufficiency (α = 0.63). Due to their low reliability, the latter two subscales were not included in the subsequent analyses of this study.

Meaning in life was assessed using the Purpose-in-Life Test-10 (PIL-10; García-Alandete et al., 2013), a shortened version of the original instrument developed by Crumbaugh and Maholick (1969) within the framework of Frankl’s logotherapy. The PIL-10 consists of 10 items rated on a 7-point Likert scale and captures two dimensions: Satisfaction and Meaning and Vital Goals and Purposes. Previous validation studies reported good internal consistency for satisfaction and meaning (α = 0.85) and acceptable reliability for vital goals and purposes (α = 0.71) (García-Alandete et al., 2013). In our sample, the PIL-10 demonstrated good internal consistency (α = 0.85 overall; α = 0.87 for satisfaction and meaning; α = 0.76 for vital goals and purposes).

Difficulties in Emotion Regulation Scale (DERS) (Gratz & Roemer, 2004; Hervás & Jódar, 2008). Emotion dysregulation was measured using the *DERS*, which conceptualizes dysregulation as deficits in emotional awareness, acceptance, modulation, and the ability to pursue goal-directed behavior under distress. For the present study, we employed the Spanish adaptation (Hervás & Jódar, 2008), further validated for adolescent populations (Gómez-Simón et al., 2014). Analyses focused specifically on the Emotional Dysregulation and Dyscontrol subscale, indexing susceptibility to overwhelming negative affect and the perception of emotions as uncontrollable and enduring. In the current sample, this subscale showed excellent internal consistency (α = 0.90).

Hopelessness was measured with the Hopelessness Scale (HS; Beck et al., 1974), a 20-item true–false instrument developed to assess negative cognitive schemas about the future. Nine items evaluate future expectations, while the remaining 11 reflect pessimistic statements. Scores range from 0 to 20, with higher scores indicating greater hopelessness. According to Beck and Steer (1988), the HS can also be clinically informative as a predictor of suicide risk. The instrument demonstrates excellent internal consistency (α = 0.93) (Beck et al., 1974). For the present study, we employed the Spanish adaptation by Aguilar et al. (1995), validated in university populations with α = 0.79 (Viñas Poch et al., 2004). In our adolescent sample, the HS demonstrated good internal consistency (α = 0.85).

### 2.3. Procedure

The study protocol was approved by the University Ethics Committee (Code UCV 2015-2016-25-V.2). Following ethical clearance, school administrators at the five participating institutions authorized data collection. Written informed consent was obtained from parents or legal guardians for underage participants, and from students aged 18 years or older. Adolescents’ assent was also obtained to ensure voluntary participation. The informed consent documents clearly described the study purpose, objectives, procedures, questionnaire duration, data confidentiality, and participants’ right to withdraw at any time without consequences.

Trained members of the research team visited each school and administered the survey on site. Participants accessed the online questionnaire (SurveyMonkey) by scanning a QR code with their mobile phones; in accordance with institutional policies restricting personal device use for students under 14 years of age (1st–2nd year of compulsory secondary education), these students completed the survey on school desktop computers. The questionnaire collected sociodemographic and psychopathological information. Approximately 1% of eligible students declined or withdrew participation, reporting that they preferred not to disclose confidential information.

### 2.4. Statistical Analysis

In the present study, descriptive statistics were computed to examine the clinical and demographic characteristics of the sample. In addition to the two participants with missing age data, a small proportion of missing values (below 3%) was observed in some study variables. Given the low level of missingness, analyses were conducted using complete cases (listwise deletion), which is considered an acceptable and widely used approach when the amount of missing data is minimal. Given the non-normal distribution of the data, Spearman’s rho correlations were used to explore the associations among the study variables. Structural equation modeling (SEM) was conducted to test the hypothesized model, which examined whether attachment styles (secure and disorganized/traumatic) were indirectly associated with MIL through two mediators: emotion dysregulation and hopelessness. Attachment styles were specified as exogenous predictors, emotion dysregulation and hopelessness were included as mediators, and two dimensions of meaning in life—satisfaction and meaning, and vital goals and purposes—were modeled as endogenous outcomes. First, we estimated a partial mediation model, in which direct paths from attachment styles to meaning in life outcomes were included along with indirect paths through the mediators. Second, we tested a trimmed model by removing non-significant paths suggested by Wald tests (e.g., direct effects from disorganized attachment to satisfaction and meaning, and from emotion dysregulation to vital goals and purposes, in order to evaluate whether a more parsimonious model would provide an equivalent or better fit. Model fit was assessed using multiple indices: the comparative fit index (CFI), the Tucker–Lewis index (TLI), the root mean square error of approximation (RMSEA) with its 90% confidence interval, and the standardized root mean square residual (SRMR). Following conventional guidelines (Hu & Bentler, 1999), values of CFI and TLI ≥ 0.95, RMSEA ≤ 0.06, and SRMR ≤ 0.08 were considered indicative of good model fit. Indirect and total effects were estimated using the delta method, and statistical significance was evaluated at *p* < 0.05. All analyses were performed using JASP (version 28.3) (JASP Team, 2023).

## 3. Results

### 3.1. Correlations

Significant correlations were observed between attachment styles and the study variables. Secure attachment correlated positively with MIL total score (*r* = 0.51, *p* < 0.001), satisfaction and meaning (*r* = 0.51, *p* < 0.001), and vital goals and purposes (*r* = 0.38, *p* < 0.001), and negatively with hopelessness (*r* = −0.45, *p* < 0.001) and emotion dysregulation (*r* = −0.39, *p* < 0.001). In contrast, disorganized attachment showed negative correlations with MIL (*r* = −0.35, *p* < 0.001), satisfaction and meaning (*r* = −0.37, *p* < 0.001), and vital goals and purposes (*r* = −0.25, *p* < 0.001), and positive correlations with hopelessness (*r* = 0.34, *p* < 0.001) and emotion dysregulation (*r* = 0.40, *p* < 0.001).

Regarding MIL, the total score correlated positively with satisfaction and meaning (*r* = 0.95, *p* < 0.001) and vital goals and purposes (*r* = 0.84, *p* < 0.001), and negatively with hopelessness (*r* = −0.64, *p* < 0.001) and emotion dysregulation (*r* = −0.47, *p* < 0.001). Both subdimensions of meaning in life showed similar patterns, with positive associations between them (*r* = 0.64, *p* < 0.001) and consistent negative associations with hopelessness and emotion dysregulation (Table 1).

### 3.2. Path Analyses

To examine the pathways linking attachment styles to MIL, a SEM was specified based on theoretical and empirical grounds. The initial model proposed that secure and disorganized attachment styles function as antecedents of two psychological vulnerabilities—emotion dysregulation and hopelessness—which in turn were hypothesized to predict satisfaction and meaning and vital goals and purposes). Covariances among mediators and between attachment styles were included to reflect their theoretical interdependence.

This initial model was just-identified (df = 0), meaning that the number of estimated parameters equaled the number of known values in the data. Consequently, fit indices reached their maximum values (e.g., CFI = 1.000, TLI = 1.000, RMSEA = 0.000, SRMR < 0.001), which precludes meaningful evaluation of overall model adequacy. The model explained 52.1% of the variance in satisfaction and meaning, 43.6% in vital goals and purposes, 26.3% in hopelessness, and 21.5% in emotion dysregulation.

As hypothesized, secure attachment was negatively associated with hopelessness (β = −0.31, *p* < 0.001) and emotion dysregulation (β = −0.37, *p* < 0.001), and positively predicted both MIL outcomes: satisfaction and meaning (β = 0.25, *p* < 0.001) and vital goals and purposes (β = 0.08, *p* < 0.001). In contrast, disorganized attachment was positively related to hopelessness (β = 0.12, *p* < 0.001) and emotion dysregulation (β = 0.49, *p* < 0.001) but showed no significant direct paths to MIL (all *p* > 0.40). Among the mediators, hopelessness strongly predicted lower satisfaction and meaning (β = −0.79, *p* < 0.001) and fewer vital goals and purposes (β = −0.64, *p* < 0.001), while emotion dysregulation significantly predicted lower satisfaction and meaning (β = −0.18, *p* < 0.001), but not vital goals and purposes (*p* = 0.133).

To further evaluate model parsimony, a trimmed model was tested by removing the non-significant direct paths from disorganized attachment to satisfaction and meaning and from emotion dysregulation to vital goals and purposes, as suggested by Wald tests. This re-specified model showed an excellent fit to the data: χ^2^(3) = 3.03, *p* = 0.388, CFI = 1.000, TLI = 1.000, RMSEA = 0.002 (90% CI [0.000, 0.037]), SRMR = 0.005. The model accounted for 52.0% of the variance in satisfaction and meaning, 43.6% in vital goals and purposes, 26.3% in hopelessness, and 21.5% in emotion dysregulation.

**Figure 1:**
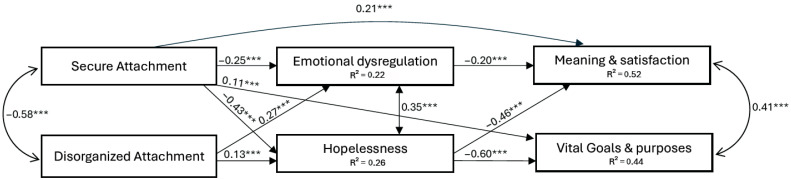
Standardized path coefficients from the structural equation model examining the relationships among attachment, emotional dysregulation, hopelessness, and meaning in life. *Note.* *** *p* < 0.001.

In this final model (Figure 1), secure attachment remained negatively associated with hopelessness (β = −0.43, *p* < 0.001) and emotion dysregulation (β = −0.25, *p* < 0.001), and positively predicted both dimensions of MIL: satisfaction and meaning (β = 0.21, *p* < 0.001) and vital goals and purposes (β = 0.11, *p* < 0.001). Disorganized attachment was positively associated with hopelessness (β = 0.13, *p* < 0.001) and emotion dysregulation (β = 0.27, *p* < 0.001) but exerted only indirect effects on MIL through these mediators. Hopelessness strongly predicted lower satisfaction and meaning (β = −0.46, *p* < 0.001) and fewer vital goals and purposes (β = −0.60, *p* < 0.001), while emotion dysregulation significantly predicted lower satisfaction and meaning (β = −0.20, *p* < 0.001), but not vital goals and purposes (*p* = 0.133) (See Table 2).

## 4. Discussion

Building on prior evidence that MIL serves as a protective factor for mental health (e.g., Brassai et al., 2011; Hill et al., 2016), this study examined how attachment may be associated with adolescents’ capacity to sustain meaning, focusing on hopelessness and emotion dysregulation as potential mediating processes. Interpreting the findings within this framework emphasizes both their theoretical relevance—clarifying mechanisms that link attachment to meaning—and their practical implications for prevention and intervention in adolescent well-being.

Regarding the first objective—examining the associations among attachment styles, emotion dysregulation, hopelessness, and MIL—the findings are consistent with prior evidence. In line with Mikulincer and Shaver’s (2013) argument that attachment security constitutes a critical basis for perceiving life as coherent, valuable, and imbued with authentic meaning, secure attachment was positively associated with both dimensions of MIL (satisfaction and meaning, as well as vital goals and purposes). This pattern suggests that attachment security may foster not only perceptions of coherence and value in life but also the motivation to pursue meaningful goals—an especially relevant process during adolescence, when individuals begin to explore autonomy and long-term aspirations (Ruiz & Yabut, 2024). By contrast, disorganized attachment was linked to lower MIL, heightened hopelessness, and greater emotion dysregulation. These results converge with Ringer et al. (2014), who documented the link between attachment insecurity and hopelessness, and further echo Main’s (1990) conceptualization of the attachment system as a regulator of distress through proximity-seeking behaviors—mechanisms that appear to be compromised in disorganized attachment and which, in adulthood, may operate through direct support seeking, the activation of internalized representations, or self-soothing routines (Mikulincer & Shaver, 2008).

Moreover, total MIL and its two dimensions were positively interrelated and negatively associated with both hopelessness, consistent with Sun et al. (2022), and emotion dysregulation, as highlighted by Lin (2022). This pattern suggests that higher levels of MIL are associated with lower cognitive and emotional vulnerability and may function as a protective correlate against such difficulties. Importantly, the findings reveal a differentiated mediational pattern across the two dimensions of MIL: satisfaction and meaning were jointly mediated by both hopelessness and emotion dysregulation, whereas the association between attachment and vital goals and purposes was primarily mediated by hopelessness. This differentiation indicates that emotion regulation strategies operating in daily life are particularly relevant for the subjective experience of coherence and meaning, consistent with theoretical models emphasizing the role of emotion regulation in shaping momentary affective experience and subjective well-being (Gross, 2015), while expectations about the future -a core component of hopelessness- are particularly decisive for sustaining purposeful projects. Such results align with Doğanülkü and Mukba (2022), while Hadley and MacLeod (2010) argue that individuals with high hopelessness do not necessarily endorse fewer goals but rather perceive them as less attainable, linking their happiness, fulfillment, and self-worth to the achievement of those specific goals. Overall, this asymmetry may reflect meaningful theoretical and developmental distinctions between the two mediators. Hopelessness, as a future-oriented cognitive–motivational construct, directly shapes expectations about goal attainability and personal agency, which may explain its stronger and more consistent mediating role, particularly for vital goals and purposes, as proposed in goal-based models of hopelessness and future thinking (Hadley & MacLeod, 2010). In contrast, emotion dysregulation captures difficulties in managing affective states in the present moment and appears more closely linked to the subjective sense that life is coherent, satisfying, and meaningful than to the maintenance of long-term projects. From a developmental perspective, during adolescence, life goals and purposes are often exploratory and unstable, rendering them especially sensitive to future-oriented beliefs rather than to momentary emotion regulation strategies, a pattern consistent with developmental models of identity and purpose formation in adolescence (Crocetti et al., 2014). Thus, while emotion dysregulation may undermine the felt sense that life is meaningful, hopelessness appears to play a more central role in shaping whether adolescents can envision and commit to purposeful life trajectories.

With respect to the second objective—examining whether attachment styles are indirectly associated with MIL through hopelessness and emotion dysregulation—the findings provide several noteworthy contributions. Secure attachment emerged as a protective factor, being directly associated with both dimensions of MIL and related to lower levels of hopelessness and emotion dysregulation. This pattern supports the view that attachment security constitutes a central resource for fostering both the perception of life as coherent and valuable (satisfaction and meaning) and the orientation toward future-directed goals and purposes (vital goals and purposes), in line with Bowlby’s (1973) propositions regarding internal working models.

In contrast, disorganized attachment did not directly predict MIL. Rather, its association with meaning in life emerged indirectly through heightened hopelessness and emotion dysregulation. Although this pattern is theoretically consistent with vulnerability-based models, it should be interpreted with caution, as the cross-sectional design precludes conclusions about temporal ordering and does not rule out alternative explanations. Notably, hopelessness emerged as the strongest mediator, exerting a pervasive negative influence on both dimensions of MIL, whereas emotion dysregulation was specifically linked to reduced satisfaction and meaning but not to vital goals and purposes. This differentiation suggests that while hopelessness undermines both the evaluative and goal-oriented facets of MIL, deficits in emotion regulation primarily compromise the capacity to experience life as coherent and meaningful. Clinically, this means that adolescents who experience high levels of hopelessness may not only struggle to find value in their present lives but also lose motivation to pursue future goals, leading to a more global sense of existential emptiness. In contrast, those with pronounced difficulties in regulating emotions may still preserve certain future aspirations but perceive their daily life as fragmented, chaotic, or lacking coherence. In practice, distinguishing between these profiles is critical, as it points to different underlying mechanisms of distress and highlights the need for tailored therapeutic approaches.

From a psychotherapeutic perspective, these results highlight the importance of addressing both vulnerabilities in adolescents with insecure or traumatic attachment histories. Interventions that target hopelessness—by strengthening optimism, self-efficacy, and adaptive goal-setting—may contribute to improvements across both dimensions of MIL, sustaining not only vital goals and purposes but also enhancing satisfaction and meaning. In parallel, programs aimed at enhancing emotion regulation skills could be especially valuable for reinforcing the evaluative facet of MIL, namely the perception of life as coherent and meaningful.

Taken together, the present findings help clarify the mechanisms through which attachment may be related to adolescents’ capacity to sustain meaning in life. Secure attachment emerged as a direct protective factor, whereas the detrimental impact of disorganized attachment was primarily associated with heightened hopelessness and difficulties in emotion regulation. The model accounted for substantial proportions of variance in both dimensions of MIL (52% in satisfaction and meaning and 44% in vital goals and purposes), highlighting the value of integrating attachment, hopelessness, and emotion dysregulation within a unified framework. These results extend previous theoretical models by positioning hopelessness and emotion dysregulation as proximal pathways linking attachment insecurity to diminished meaning in life.

### Limitations, Future Directions, and Clinical Implications

This study is subject to some limitations that should be acknowledged. The cross-sectional design prevents causal inference and raises the possibility that the associations observed may be bidirectional. Exclusive reliance on self-report instruments may also have introduced common method variance and response biases, particularly given that attachment, hopelessness, and emotion regulation are constructs highly sensitive to social desirability and retrospective distortion. In this context, the indirect effects observed for disorganized attachment may also reflect shared method variance or the influence of unmeasured third variables, which cannot be disentangled within a cross-sectional design. Furthermore, the use of a convenience sample drawn from a limited number of schools in three Spanish regions constrains the generalizability of the findings, which should not be assumed to extend to other countries or sociocultural contexts, where attachment-related processes and meaning construction may differ.

Despite these limitations, the study makes a novel contribution by integrating attachment, hopelessness, and emotion dysregulation into a unified explanatory model of MIL in adolescence. Future research should prioritize longitudinal designs—such as latent growth modeling or cohort studies—that allow testing of causal pathways and developmental trajectories. Incorporating multi-method approaches that combine self-reports with behavioral observations or informant ratings would reduce bias and strengthen construct validity. Extending recruitment to more culturally and geographically diverse samples is also essential for testing the cross-cultural generalizability of these mechanisms.

Clinically, the findings underscore the importance of addressing hopelessness and emotion dysregulation in adolescents with insecure or traumatic attachment histories. Interventions that explicitly target these vulnerabilities may safeguard adolescents’ capacity to construct and maintain a sense of meaning, thereby mitigating broader psychopathological risks. Among evidence-based approaches, Mentalization-Based Treatment for Adolescents (MBT-A) has shown promise in enhancing reflective functioning and improving attachment-related processes (Rossouw & Fonagy, 2012), while Dialectical Behavior Therapy for Adolescents (DBT-A) is particularly relevant for those exhibiting severe emotional dysregulation or behavioral dyscontrol, offering structured skills training in emotion regulation, distress tolerance, and interpersonal effectiveness (Miller et al., 2007). Rather than viewing these interventions as interchangeable, the present findings suggest that tailoring strategies to address the specific vulnerabilities identified—hopelessness as the most robust mediator and emotion dysregulation as a secondary but significant factor—may be especially effective. Advancing research along these lines will help refine theoretical models of MIL and guide the development of targeted interventions. Ultimately, strengthening adolescents’ capacity to sustain meaning through meaning-based interventions (Breitbart & Poppito, 2014) may serve as a cornerstone of mental health promotion, bridging developmental theory with applied clinical practice.

## Figures and Tables

**Table 1:** Correlations between the study variables and descriptive statistics.

Variable	1	2	3	4	5	6	7	*M*	*SD*
1. Secure attachment	—							29.67	5.65
2. Disorganized attachment	−0.521 ***	—						8.88	4.60
3. Meaning in life (total)	0.507 ***	−0.350 ***	—					48.80	10.57
4. Meaning and satisfaction	0.511 ***	−0.368 ***	0.947 ***	—				27.50	7.08
5. Vital goals and purposes	0.379 ***	−0.246 ***	0.844 ***	0.639 ***	—			21.31	4.41
6. Hopelessness	−0.448 ***	0.337 ***	−0.642 ***	−0.593 ***	−0.575 ***	—		4.52	4.07
7. Emotion dysregulation	−0.385 ***	0.404 ***	−0.469 ***	−0.482 ***	−0.333 ***	0.452 ***	—	20.09	8.29

Note. Spearman’s rho correlations. *** *p* < 0.001 (two-tailed).

**Table 2:** Model fit indices for the SEM models.

Mode	χ^2^ (df), *p*	CFI	TLI	RMSEA [90% CI]	SRMR	AIC	BIC	R^2^ (Life Satisfaction & Sense)	R^2^ (Life Goals)	R^2^ (Hope-Lessness)	R^2^ (Emotion Dysregulation)
Initial (just-identified)	— (0), —	1.000	1.000	0.000 [0.000, 0.000] *	<0.001	70,546.689	70,698.593	0.521	0.436	0.263	0.215
Trimmed (final)	3.03 (3), *p* = 0.388	1.000	1.000	0.002 [0.000, 0.037]	0.005	70,543.714	70,678.740	0.520	0.436	0.263	0.215

*Note.* The initial model is just-identified (df = 0); χ^2^, RMSEA, CFI/TLI are not informative in this case. We report AIC/BIC and R^2^ for completeness. Lower AIC/BIC indicate better relative fit; the trimmed model shows slightly lower values (better parsimony). Statistical symbols are italicized in text, not in tables (APA 7). * *p* < 0.05.

## Data Availability

The data presented in this study are openly available in [Harvard Dataverse] at [https://doi.org/10.7910/DVN/YVHAXK], accessed on 3 February 2026.
